# Development and internal validation of a LASSO-based clinical prediction model for nontuberculous mycobacterial pulmonary disease versus pulmonary tuberculosis

**DOI:** 10.3389/fmed.2026.1785899

**Published:** 2026-04-01

**Authors:** Haiqing Liu, Mingfeng Han, Guoling Cheng, Hao Yan, Jing Hou, Xiaoyu Cao, Wei Zhang

**Affiliations:** The Second People's Hospital of Fuyang City, Fuyang Infection Disease Clinical College of Anhui Medical University, Fuyang, Anhui, China

**Keywords:** differential diagnosis, LASSO regression, nontuberculous mycobacterial lung disease, prediction model, pulmonary tuberculosis

## Abstract

**Background:**

The rising global incidence of nontuberculous mycobacterial pulmonary disease (NTM-PD) and its significant overlap with pulmonary tuberculosis (PTB) in symptoms and imaging pose a major diagnostic challenge, often leading to misdiagnosis and inappropriate treatment. A reliable pre-culture predictive tool is urgently needed.

**Methods:**

In this retrospective cross-sectional study, we analyzed consecutive hospitalized patients with microbiologically confirmed NTM-PD (*n* = 145) or PTB (*n* = 206) from January 2021 to December 2023. Demographic, clinical, comorbidity, laboratory, and high-resolution CT (HRCT) data were collected. Least Absolute Shrinkage and Selection Operator (LASSO) regression with 10-fold cross-validation was used for feature selection. Selected variables were incorporated into a multivariate logistic regression model to construct a final prediction model. Model performance was evaluated by area under the receiver operating characteristic curve (AUC), calibration (Hosmer-Lemeshow test, calibration plot), and internal validation via 1,000 bootstrap resamples. Clinical utility was assessed using decision curve analysis (DCA).

**Results:**

The LASSO regression identified six independent predictors for the final model: older age, female gender, absence of diabetes mellitus, presence of bronchiectasis, presence of chronic obstructive pulmonary disease (COPD), and presence of lung cavitation on HRCT. The model demonstrated good discrimination with an AUC of 0.846 (95% CI, 0.805–0.877) and excellent calibration (Hosmer-Lemeshow test, *p* = 0.949). Bootstrap internal validation yielded an optimism-corrected concordance index of 0.830. DCA confirmed the model’s clinical net benefit across a wide range of threshold probabilities.

**Conclusion:**

We developed and internally validated a parsimonious six-variable prediction model that effectively differentiates NTM-PD from PTB. Incorporating objective feature selection (LASSO) and rigorous validation, this tool can aid clinicians in raising early suspicion for NTM-PD, optimizing diagnostic pathways, and preventing misdiagnosis while awaiting culture results.

## 1 Introduction

The global burden of nontuberculous mycobacterial pulmonary disease (NTM-PD) is rising at an alarming rate. A recent systematic review and meta-analysis demonstrated that the incidence of pulmonary NTM infection and disease is increasing worldwide, with an estimated annual rate of change of +4.0% and +4.1% per 100,000 persons/year, respectively (1). This burden is particularly pronounced in specific populations; for instance, in East Asia, North America, and Australia, NTM-PD incidence and prevalence remain generally higher than in Europe (2). Nationwide surveillance data from China between 2013 and 2017 showed that NTM accounted for 17.8% of culture-positive mycobacterial isolates, with an estimated incidence of 4.1 per 100,000 population, rising to 11.5 per 100,000 in elderly women (3). This escalating epidemic poses a formidable clinical challenge, especially in tuberculosis (TB)-endemic regions, due to the profound symptomatic and radiographic overlap between NTM-PD and pulmonary tuberculosis (PTB) (4, 5). Shared features such as chronic cough, fever, and hemoptysis, alongside imaging findings like nodules and cavities, frequently lead to diagnostic confusion (6).

Definitive diagnosis hinges on microbiological confirmation, which is hampered by prolonged culture times, susceptibility to contamination, and limited accessibility in resource-constrained settings (7). Consequently, misdiagnosis is common, often resulting in inappropriate anti-tuberculosis treatment that contributes to treatment failure, adverse effects, and antimicrobial resistance (8, 9). Thus, there is a pressing need for accurate, pre-culture clinical tools to differentiate NTM-PD from PTB.

Previous studies have identified potential discriminators, including older age, female sex, underlying bronchiectasis or COPD, and specific CT patterns (10–12). However, many existing models suffer from methodological limitations: they often rely on univariate screening without robust multivariable integration, use conventional stepwise regression prone to overfitting, or lack rigorous internal validation (13, 14). This compromises their generalizability and clinical utility.

To overcome these shortcomings, we aimed to develop and validate a clinically actionable prediction model using advanced statistical techniques. We incorporated readily available clinical and radiographic data. Methodologically, our study advances the field by: (1) applying LASSO regression for automated, penalized feature selection to enhance model parsimony and address multicollinearity; (2) developing a multivariate logistic model; and (3) implementing comprehensive validation via bootstrap resampling and decision curve analysis (DCA) to rigorously assess performance and clinical net benefit. We hypothesize that this approach will yield a more reliable and deployable tool for distinguishing NTM-PD from PTB in routine practice.

## 2 Methods

### 2.1 Study design and participants

This retrospective cross-sectional study was conducted at the Second People’s Hospital of Fuyang City. We systematically reviewed the electronic medical records of all consecutive patients hospitalized between January 1, 2021, and December 31, 2023, with a suspected or confirmed diagnosis of mycobacterial pulmonary disease.

Study Groups: Patients were classified into two groups based on their final microbiological diagnosis: (1) the NTM-PD group (*n* = 145) and (2) the PTB group (*n* = 206), which served as the comparator group.

Inclusion Criteria: (1) Age ≥ 18 years; (2) A definitive diagnosis of either NTM-PD or PTB based on microbiological confirmation during the index hospitalization. The reference standards for diagnosis are detailed in Section 2.3.

Exclusion Criteria: Patients were excluded if they met any of the following conditions: (1) Co-infection with both NTM and *Mycobacterium tuberculosis* (*n* = 8); (2) Diagnosis of lung cancer, other active malignancies, or human immunodeficiency virus (HIV) infection (*n* = 12); (3) Presence of other active pulmonary infections (e.g., fungal pneumonia, severe community-acquired pneumonia) that could confound radiographic or clinical assessment (*n* = 8); (4) Incomplete clinical, imaging, or laboratory records precluding comprehensive analysis (*n* = 3).

**Figure 1:**
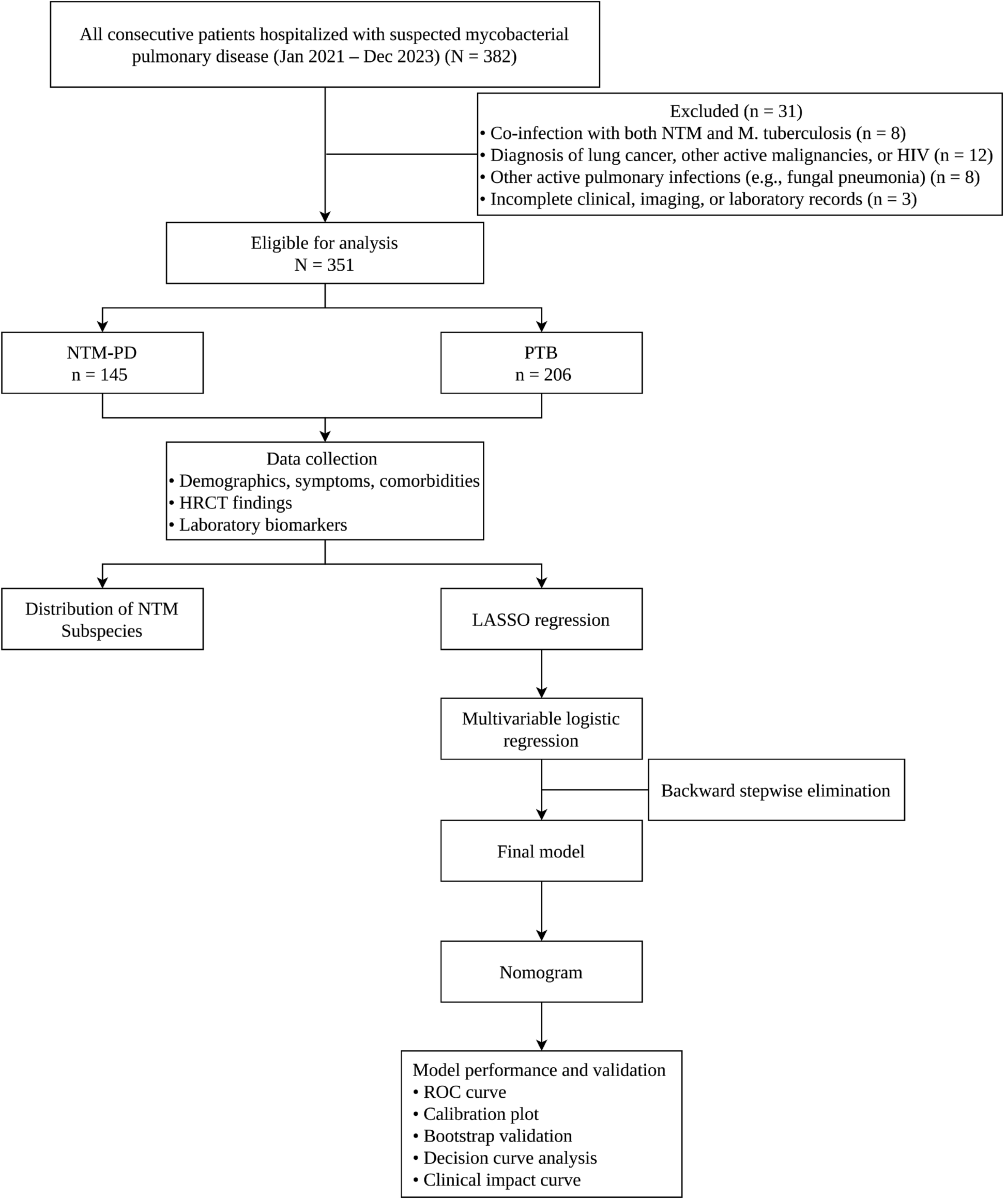
Study workflow diagram. The flowchart illustrates the entire study process. Initially, 382 hospitalized patients with suspected mycobacterial pulmonary disease were screened. After applying exclusion criteria (co-infection, malignancies/HIV, other active pulmonary infections, incomplete records), 351 patients were enrolled, comprising 145 NTM-PD cases and 206 PTB controls. Data on demographics, clinical symptoms, comorbidities, HRCT findings, and laboratory biomarkers were collected. All candidate variables were then subjected to LASSO regression with 10-fold cross-validation for feature selection. Multivariable logistic regression with backward stepwise elimination yielded the final model. Model performance was assessed by discrimination (AUC), calibration (Hosmer-Lemeshow test), internal validation (1,000 bootstrap resamples), and clinical utility (decision curve analysis).

Following the application of these criteria, a total of 351 patients were included in the final analysis. The patient screening and inclusion process, along with the subsequent steps of model development and validation, is detailed in the study workflow diagram (Figure 1).

### 2.2 Ethical considerations

The entire research process was conducted in strict accordance with the ethical principles outlined in the Declaration of Helsinki and its subsequent amendments, ensuring the ethical legitimacy of the study. The study protocol was thoroughly reviewed and formally approved by the Institutional Ethics Committee of the Second People’s Hospital of Fuyang City (Approval No.: 20211231014). Due to the retrospective nature of the study, which involved the analysis of anonymized data from existing medical records, the requirement for informed consent was waived by the aforementioned Ethics Committee.

### 2.3 Diagnostic criteria

Reference standard for NTM-PD: Diagnosis was established according to the 2020 Chinese guidelines for NTM diseases (15), which align with the core principles of the American Thoracic Society/Infectious Diseases Society of America (ATS/IDSA) guidelines (16). This required: (1) clinical and radiographic signs suggestive of pulmonary disease, and (2) microbiological confirmation defined as at least two separate positive cultures for NTM from sputum samples, or one positive culture from bronchoalveolar lavage fluid (BALF) or lung biopsy tissue. All NTM isolates were identified to the species/complex level using molecular methods (e.g., DNA sequencing or PCR-based hybridization).

Reference standard for PTB: Diagnosis was made in accordance with the Chinese national standard (WS 288–2017) (17), requiring microbiological confirmation defined as either: (1) positive culture for *M. tuberculosis*, or (2) a positive acid-fast bacilli (AFB) smear combined with compatible clinical and radiographic findings and a subsequent response to anti-tuberculosis therapy.

### 2.4 Data collection and variable definitions

Demographic, clinical, comorbidity, and laboratory data were extracted from the hospital’s electronic health record system by two trained researchers using a standardized case report form. Any discrepancies were resolved by consensus or adjudication by a senior pulmonologist.

Clinical symptoms: Symptoms present at admission were recorded as dichotomous variables (Yes/No). The severity of dyspnea was graded using the modified Medical Research Council (mMRC) dyspnea scale (18), but for the primary model, dyspnea was analyzed as present (mMRC grade ≥1) or absent. Sputum production was categorized as present or absent based on patient report and clinical documentation.

Comorbidities: Diagnoses of bronchiectasis, emphysema, and chronic obstructive pulmonary disease (COPD) were confirmed based on prior clinical documentation and corroborative imaging (HRCT for bronchiectasis and emphysema) or spirometry where available. Diabetes mellitus and hypertension were defined by a documented diagnosis or ongoing pharmacologic treatment.

### 2.5 Radiographic assessment

All patients underwent non-contrast high-resolution computed tomography (HRCT) of the chest within 1 week of admission. Imaging findings were assessed independently by two experienced thoracic radiologists who were blinded to the patients’ final microbiological diagnosis. They evaluated the presence or absence of predefined radiographic patterns: patchy infiltrates, nodular lesions, cystic bronchiectasis, linear opacities, ground-glass opacities, honeycombing, fibrotic changes, and lung cavitation. Any disagreement was discussed to reach a consensus. The inter-reader reliability for key findings (e.g., cavitation, bronchiectasis) was assessed using Cohen’s kappa statistic, with values interpreted according to recent guidelines for diagnostic agreement studies (19).

### 2.6 Laboratory measurements

Fasting venous blood samples were collected within 24 h of admission. Serum levels of C-reactive protein (CRP) and serum amyloid A (SAA) were measured using a Hitachi 7,600 automated biochemistry analyzer. Complete blood counts, including white blood cell (WBC) count, neutrophil percentage (NEUT%), lymphocyte percentage (LYMPH%), and platelet count (PLT), were determined using a Sysmex XE-2100 automated hematology analyzer. Derived ratios, including neutrophil-to-lymphocyte ratio (NLR), platelet-to-lymphocyte ratio (PLR), and SAA/CRP ratio, were calculated.

### 2.7 Statistical analysis

Statistical analyses were performed using R software (version 4.3.0) and IBM SPSS Statistics (version 26.0). A two-sided *p*-value < 0.05 was considered statistically significant.

Descriptive Statistics: Continuous variables were tested for normality using the Shapiro–Wilk test. Normally distributed data are presented as mean ± standard deviation (SD) and compared using Student’s *t*-test. Non-normally distributed data are presented as median with interquartile range (IQR) and compared using the Mann–Whitney *U* test. Categorical variables are presented as frequencies (percentages) and compared using the Chi-square test or Fisher’s exact test, as appropriate.

Feature Selection and Model Development: To address multicollinearity and select the most relevant predictors from all candidate variables, Least Absolute Shrinkage and Selection Operator (LASSO) regression with 10-fold cross-validation was applied. The optimal tuning parameter (*λ*) was chosen based on the 1-standard error criterion (lambda.1se), which selects the most parsimonious model whose cross-validation error is within one standard error of the minimum. Variables with non-zero coefficients at lambda.1se were retained for subsequent multivariable analysis. To ensure reproducibility, a random seed was set (seed = 1,234).

Multivariable Logistic Regression: The variables selected by LASSO were entered into a binary logistic regression model with NTM-PD as the dependent variable. A stepwise backward elimination procedure (removal criterion: *p* > 0.05) was then used to derive a parsimonious final prediction model. The results are presented as odds ratios (ORs) with 95% confidence intervals (CIs). Variance inflation factors (VIFs) were examined to confirm the absence of significant multicollinearity (all VIF < 2.5) in the final model.

Model Performance and Validation: The discriminatory ability of the final model was evaluated by the area under the receiver operating characteristic curve (AUC). The optimal probability cut-off point for classification was determined using the Youden index (20), which maximizes the sum of sensitivity and specificity. Calibration was assessed using the Hosmer-Lemeshow goodness-of-fit test and a calibration plot. Internal validation was performed using the bootstrap resampling method with 1,000 iterations using a fixed random seed (seed = 123) to obtain an optimism-corrected concordance index (C-index), providing a more robust and reproducible estimate of model performance. Decision curve analysis (DCA) was conducted to evaluate the clinical net benefit of the model across a range of probability thresholds.

## 3 Results

### 3.1 Baseline characteristics of the study population

Significant intergroup differences (*p* < 0.05) were observed across multiple domains (Table 1). Compared to the PTB group, patients with NTM-PD were older, had a lower BMI, and included a higher proportion of females and farmers. Symptoms of dyspnea, loss of appetite, and fever were more prevalent in the NTM-PD cohort. A distinct comorbidity profile was noted: diabetes mellitus was less frequent, while bronchiectasis, emphysema, and COPD were more common in NTM-PD patients. Radiographically, cystic bronchiectasis, honeycombing, and lung cavitation were more frequently associated with NTM-PD. In contrast, no significant differences were found in the majority of inflammatory biomarkers.

**Table 1:** Baseline characteristics of patients with NTM-PD versus PTB.

Characteristic	NTM-PD (*n* = 145)	PTB (*n* = 206)	*P*-value
Demographics			
Age, years [M(IQR)]	68 (59,75)	59.5 (44.75,71)	<0.001
Gender [*n* (%)]			0.034
Male	94 (64.83)	155 (75.24)	
Female	51 (35.17)	51 (24.76)	
Occupation, *n* (%)			0.010^a^
Farmer	128 (88.28)	154 (74.76)	
Office worker	7 (4.83)	18 (8.74)	
Retired	5 (3.45)	7 (3.40)	
Student	0 (0.00)	7 (3.40)	
Other^*^	5 (3.45)	20 (9.71)	
BMI, kg/m^2^ (mean ± SD)	18.71 ± 2.70	20.41 ± 3.50	<0.001
Clinical symptoms			
Cough, *n* (%)	135 (93.10)	181 (87.86)	0.107
Sputum production, *n* (%)	129 (88.97)	169 (82.04)	0.353
Hemoptysis, *n* (%)	28 (19.31)	31 (15.05)	0.293
Dyspnea, *n* (%)	92 (63.45)	74 (35.92)	<0.001
Loss of appetite, *n* (%)	77 (53.10)	60 (29.13)	<0.001
Fever, *n* (%)	75 (51.72)	77 (37.38)	0.008
Risk factors and comorbidities			
Smoking history, *n* (%)	52 (35.86)	96 (46.60)	0.045
Alcohol consumption history, *n* (%)	35 (24.14)	76 (36.89)	0.011
Diabetes mellitus, *n* (%)	12 (8.28)	57 (27.67)	<0.001
Hypertension, *n* (%)	17 (11.72)	33 (16.02)	0.257
Bronchiectasis, *n* (%)	42 (28.97)	11 (5.34)	<0.001
Emphysema, *n* (%)	18 (12.41)	11 (5.34)	0.018
COPD, *n* (%)	43 (29.66)	13 (6.31)	<0.001
Radiographic features (HRCT)			
Patchy infiltrates, *n* (%)	126 (86.90)	189 (91.75)	0.140
Nodular lesions, *n* (%)	68 (46.90)	112 (54.37)	0.168
Cystic bronchiectasis, *n* (%)	17 (11.72)	6 (2.91)	0.001
Linear opacities, *n* (%)	65 (44.83)	72 (34.95)	0.062
Ground-glass opacities, *n* (%)	5 (3.45)	3 (1.46)	0.283^a^
Honeycombing patterns, *n* (%)	9 (6.21)	2 (0.97)	0.009^a^
Fibrotic changes, *n* (%)	2 (1.38)	0 (0.00)	0.170^a^
Lung cavitation, *n* (%)	107 (73.79)	97 (47.09)	<0.001
Inflammatory biomarkers			
CRP, mg/L [M(IQR)]	35.50 (8.75, 78.90)	23.55 (5.35, 57.75)	0.072
SAA, mg/L [M(IQR)]	64.50 (23.35, 149.10)	50.90 (14.38, 109.98)	0.112
WBC, ×10^9^/L [M(IQR)]	6.90 (5.56, 9.28)	6.77 (5.12, 8.60)	0.166
Neutrophil percentage, % (mean ± SD)	67.70 ± 11.77	65.72 ± 12.23	0.130
Lymphocyte percentage, % [M(IQR)]	19.00 (12.05, 27.30)	20.30 (14.08, 27.53)	0.457
Neutrophil count, ×10^9^/L [M(IQR)]	4.68 (3.33, 6.77)	4.36 (3.08, 6.20)	0.117
Lymphocyte count, ×10^9^/L [M(IQR)]	1.32 (0.92,1.79)	1.30 (0.94,1.74)	0.822
Platelet count, ×10^9^/L (mean ± SD)	276.99 ± 106.10	282.58 ± 110.16	0.635
SAA/CRP ratio [M(IQR)]	1.89 (0.94,4.32)	2.26 (0.99,4.40)	0.444
NLR [M(IQR)]	3.70 (2.10,6.23)	3.30 (2.07,5.42)	0.350
PLR [M(IQR)]	203.28 (136.60, 295.80)	198.26 (139.50, 315.82)	0.543

^*^Other occupation includes self-employed and freelance workers.

^a^Fisher’s exact test was used for categorical variables with expected frequencies <5; otherwise, Chi-square test was used for categorical variables and Mann–Whitney *U* test for continuous variables.

Data are presented as median (interquartile range, IQR) for continuous variables and number (percentage) for categorical variables.

BMI, body mass index; COPD, chronic obstructive pulmonary disease; CRP, C-reactive protein; HRCT, high-resolution computed tomography; M, median; NLR, neutrophil-to-lymphocyte ratio; NTM-PD, nontuberculous mycobacterial pulmonary disease; PLR, platelet-to-lymphocyte ratio; PTB, pulmonary tuberculosis; SAA, serum amyloid A; WBC, white blood cell count.

The complete results of all descriptive statistics and comparative analyses—including Student’s t-test, Mann–Whitney *U* test, Chi-square test, and Fisher’s exact test—are presented in Table 1.

### 3.2 Distribution of nontuberculous mycobacteria subspecies

The distribution of NTM subspecies among the 145 NTM-PD patients is detailed in Table 2. The most prevalent subspecies was *Mycobacterium avium* complex (MAC), accounting for 46.21% (*n* = 67) of cases, followed by *Mycobacterium abscessus* (26.90%, *n* = 39) and *Mycobacterium kansasii* (19.31%, *n* = 28). The remaining 11 cases (7.58%) were caused by other NTM subspecies.

**Table 2:** Distribution of NTM subspecies among patients with NTM-PD (*n* = 145).

NTM subspecies	Number of cases	Proportion (%)
*Mycobacterium avium* complex (MAC)	67	46.21
*Mycobacterium abscessus*	39	26.90
*Mycobacterium kansasii*	28	19.31
Other subspecies	11	7.58
Total	145	100

Other subspecies included *M. fortuitum* (*n* = 4), *M. gordonae* (*n* = 3), *M. chelonae* (*n* = 2), *M. mucogenicum* (*n* = 1), and *M. szulgai* (*n* = 1).

### 3.3 Feature selection and development of the prediction model

**Figure 2:**
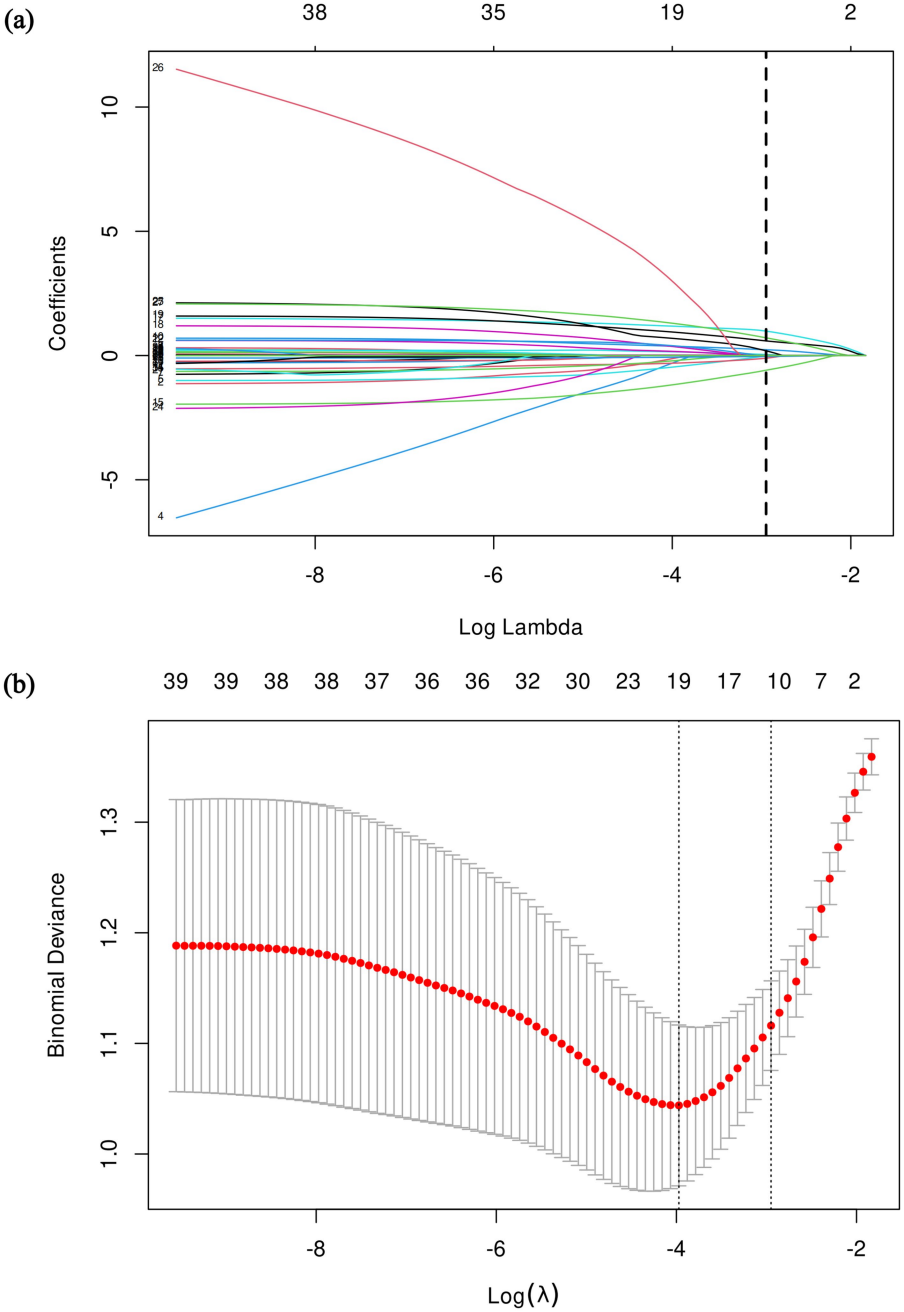
Clinical feature selection using LASSO regression models. **(a)** LASSO coefficient paths for all candidate variables. Each colored line represents the trajectory of a variable’s coefficient as *λ* increases. The vertical dashed line marks the optimal *λ* (*λ* = 0.052) selected by 10-fold cross-validation based on the 1-standard error criterion (lambda.1se). At this *λ* value, 11 variables had non-zero coefficients and were retained for further analysis. **(b)** Cross-validation plot for tuning parameter (*λ*) selection. The red dots represent the mean binomial deviance for each *λ* value across 10-fold cross-validation, and the error bars indicate the corresponding confidence intervals. The left vertical dashed line indicates the λ value corresponding to the minimum deviance (*λ*_min), and the right vertical dashed line indicates the *λ* value corresponding to the 1-standard error criterion (*λ*_1se).

To develop a clinical prediction model for differentiating NTM-PD from PTB, Least Absolute Shrinkage and Selection Operator (LASSO) regression was employed for feature selection (Figures 2a,b). Based on the 1-standard error criterion (lambda.1se) in 10-fold cross-validation, the optimal tuning parameter was determined at *λ* = 0.052. At this λ value, 11 variables with non-zero coefficients were retained for subsequent modeling: Age, Gender, BMI, Dyspnea, Loss of appetite, Alcohol consumption history, Diabetes mellitus, Bronchiectasis, COPD, Honeycombing patterns, and Lung cavitation.

### 3.4 Development and visualization of the multivariable prediction model

The 11 candidate predictors selected by LASSO regression were entered into a multivariable binary logistic regression analysis. Table 3 presents the preliminary results of this analysis, including all 11 variables. Following a stepwise backward elimination procedure (removal criterion: *p* > 0.05), a parsimonious final model was derived, retaining six independent predictors that remained statistically significant: Age, Gender, Diabetes mellitus, Bronchiectasis, COPD, and Lung cavitation. Table 4 presents the results of this final model.

**Table 3:** Initial LASSO screening and preliminary multivariate regression analysis.

Variable	*β*	SE	Wald *χ*^2^	*p*-value	OR (95%CI)
Age	0.025	0.009	2.716	0.007	1.025 (1.007 ~ 1.043)
Gender					
Female					1.000 (Reference)
Male	−0.740	0.365	−2.024	0.043	0.477 (0.233 ~ 0.977)
BMI	−0.074	0.046	−1.600	0.110	0.929 (0.849 ~ 1.017)
Dyspnea					
No					1.000 (Reference)
Yes	0.456	0.293	1.560	0.119	1.578 (0.890 ~ 2.800)
Loss of appetite					
No					1.000 (Reference)
Yes	0.237	0.302	0.783	0.434	1.267 (0.700 ~ 2.292)
Alcohol consumption history					
No					1.000 (Reference)
Yes	−0.510	0.337	−1.512	0.131	0.601 (0.310 ~ 1.163)
Diabetes mellitus					
No					1.000 (Reference)
Yes	−1.661	0.427	−3.889	<0.001	0.190 (0.082 ~ 0.439)
Bronchiectasis					
No					1.000 (Reference)
Yes	1.708	0.443	3.857	<0.001	5.516 (2.316 ~ 13.135)
COPD					
No					1.000 (Reference)
Yes	1.187	0.419	2.835	0.005	3.279 (1.443 ~ 7.450)
Honeycombing patterns					
No					1.000 (Reference)
Yes	1.548	0.927	1.671	0.095	4.703 (0.765 ~ 28.918)
Lung cavitation					
No					1.000 (Reference)
Yes	1.725	0.309	5.586	<0.001	5.611 (3.063 ~ 10.276)

This table presents the results of multivariable logistic regression including all 11 variables selected by LASSO. Variables with *P* > 0.05 were subsequently eliminated in the stepwise backward procedure to derive the final model (see Table 4).

CI, confidence interval; COPD, chronic obstructive pulmonary disease; OR, odds ratio; SE, standard error.

**Table 4:** Results of multifactorial logistic regression analysis of the final prediction model.

Variable	β	SE	Wald *χ*^2^	*P*-value	OR (95%CI)	VIF
Age	0.031	0.009	3.517	<0.001	1.031 (1.014 ~ 1.049)	1.091
Gender						1.095
Female					1.000 (Reference)	
Male	−1.094	0.323	−3.393	<0.001	0.335 (0.178 ~ 0.630)	
Diabetes mellitus						1.045
No					1.000 (Reference)	
Yes	−1.883	0.405	−4.654	<0.001	0.152 (0.069 ~ 0.336)	
Bronchiectasis						1.104
No					1.000 (Reference)	
Yes	1.694	0.428	3.962	<0.001	5.443 (2.354 ~ 12.584)	
COPD						1.153
No					1.000 (Reference)	
Yes	1.460	0.399	3.661	<0.001	4.308 (1.971 ~ 9.415)	
Lung cavitation						1.055
No					1.000 (Reference)	
Yes	1.794	0.303	5.929	<0.001	6.011 (3.322 ~ 10.875)	

This table presents the final model after backward stepwise elimination, retaining six independent predictors (all *P* < 0.001). Variance inflation factors (VIFs) confirm the absence of significant multicollinearity (all VIF < 2.5).

CI, confidence interval; COPD, chronic obstructive pulmonary disease; OR, odds ratio; SE, standard error; VIF, variance inflation factor.

**Figure 3:**
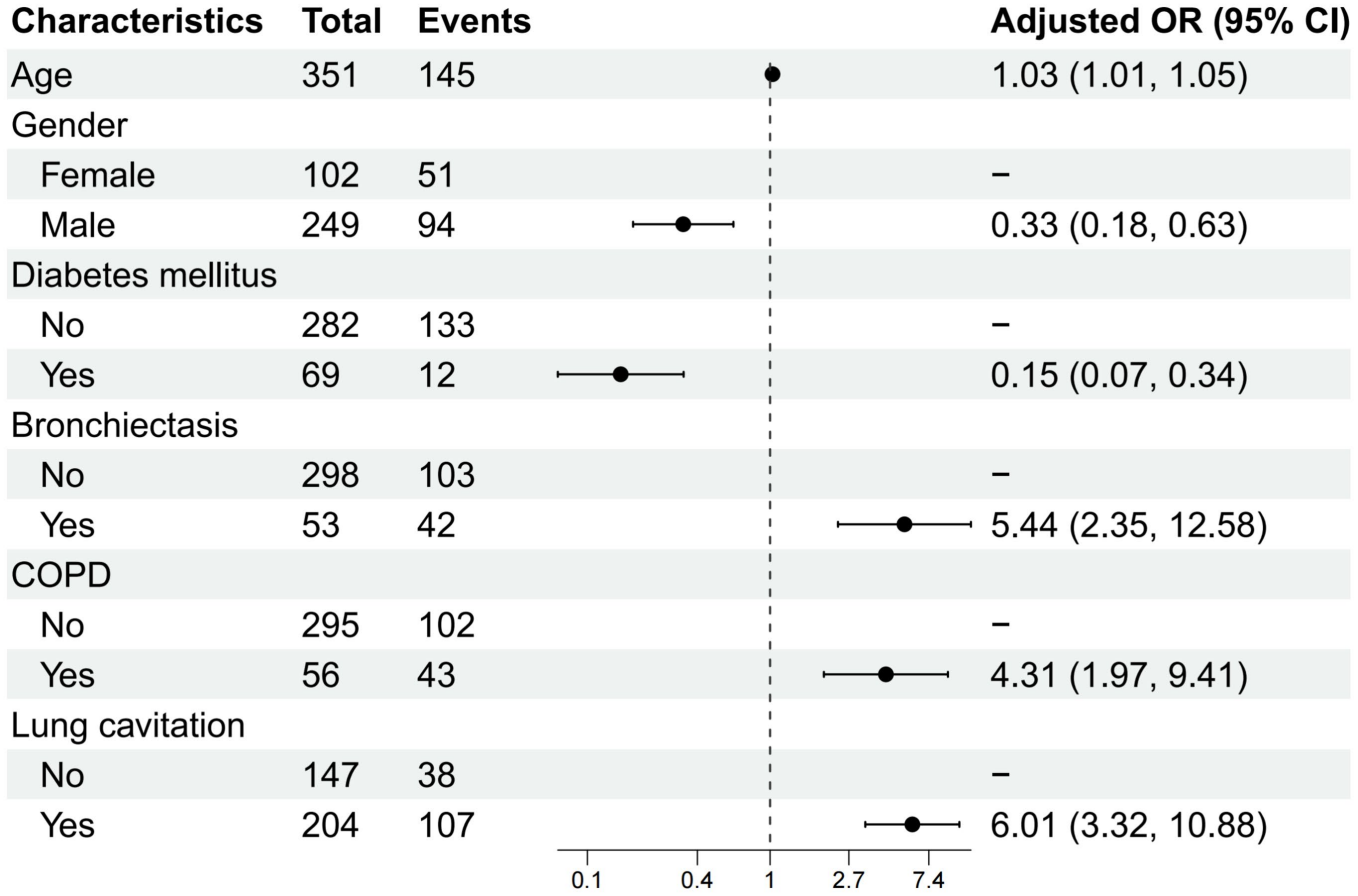
Forest plot of the final prediction model for NTM-PD. The plot shows the odds ratios (ORs) and 95% confidence intervals (CIs) for the six independent predictors retained in the final multivariable logistic regression model. NTM-PD was coded as the event of interest (dependent variable = 1), with PTB as the reference category (dependent variable = 0). Therefore, OR > 1 indicates higher odds of NTM-PD; OR < 1 indicates lower odds of NTM-PD (i.e., higher odds of PTB). Dots represent point estimates, horizontal lines represent 95% CIs, and the vertical dashed line at OR = 1 indicates no effect. Age was analyzed as a continuous variable (OR per year increase).

In this final model (Figure 3), older age (OR per year = 1.031, 95% CI: 1.014–1.049), bronchiectasis (OR = 5.443, 95% CI: 2.354–12.584), COPD (OR = 4.308, 95% CI: 1.971–9.415), and lung cavitation on HRCT (OR = 6.011, 95% CI: 3.322–10.875) were associated with higher odds of NTM-PD. Conversely, male gender (OR = 0.335, 95% CI: 0.178–0.630) and absence of diabetes mellitus (OR = 0.152, 95% CI: 0.069–0.336) were associated with lower odds of NTM-PD.

It should be noted that the forest plot presents the odds ratios (ORs) for predictors associated with NTM-PD relative to PTB. In this binary logistic regression model, NTM-PD was coded as the event of interest (dependent variable = 1) and PTB served as the reference category (dependent variable = 0). Therefore, the model directly estimates the probability of NTM-PD, and the coefficients represent the change in log-odds of NTM-PD versus PTB for each predictor. The forest plot is presented only for NTM-PD because PTB is the implicit reference group; its contribution to the model is captured in the intercept term, which is not typically displayed in forest plots for logistic regression.

**Figure 4:**
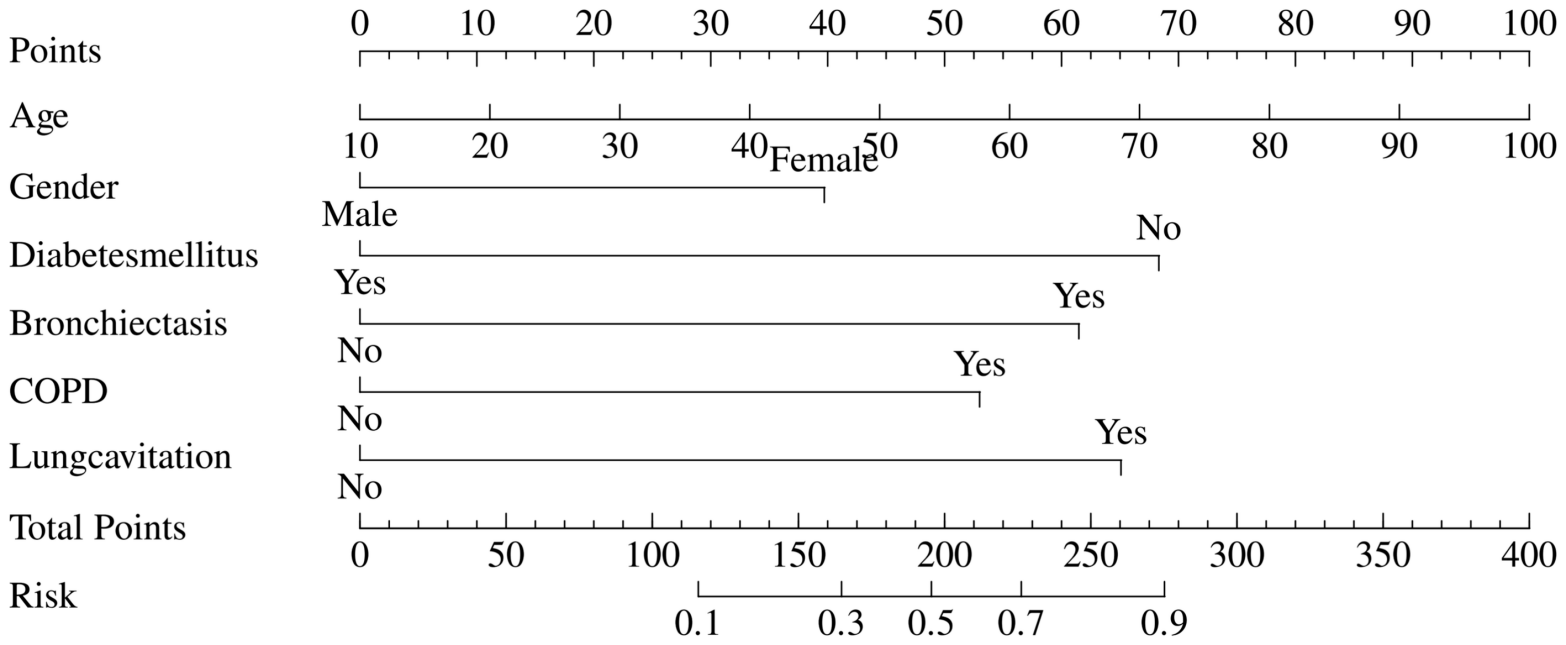
Nomogram for predicting the probability of NTM-PD based on the six-predictor logistic regression model. To use the nomogram, locate the patient’s value for each predictor on the corresponding axis. Draw a vertical line upward to the “Points” scale to obtain the points assigned for that predictor. Sum the points for all six predictors, and find this total on the “Total Points” axis. Finally, draw a vertical line downward from the “Total Points” axis to the “Risk” axis to obtain the individual predicted probability. Age is scored as a continuous variable; gender, diabetes mellitus, bronchiectasis, COPD, and lung cavitation are scored as binary variables.

To facilitate practical clinical use, a nomogram was developed based on this final six-predictor model (Figure 4). The nomogram allows clinicians to assign points for each predictor (including a continuous score for Age) and sum them to obtain a total points score, which corresponds to an individualized probability of the patient having NTM-PD.

### 3.5 Model performance and validation

**Figure 5:**
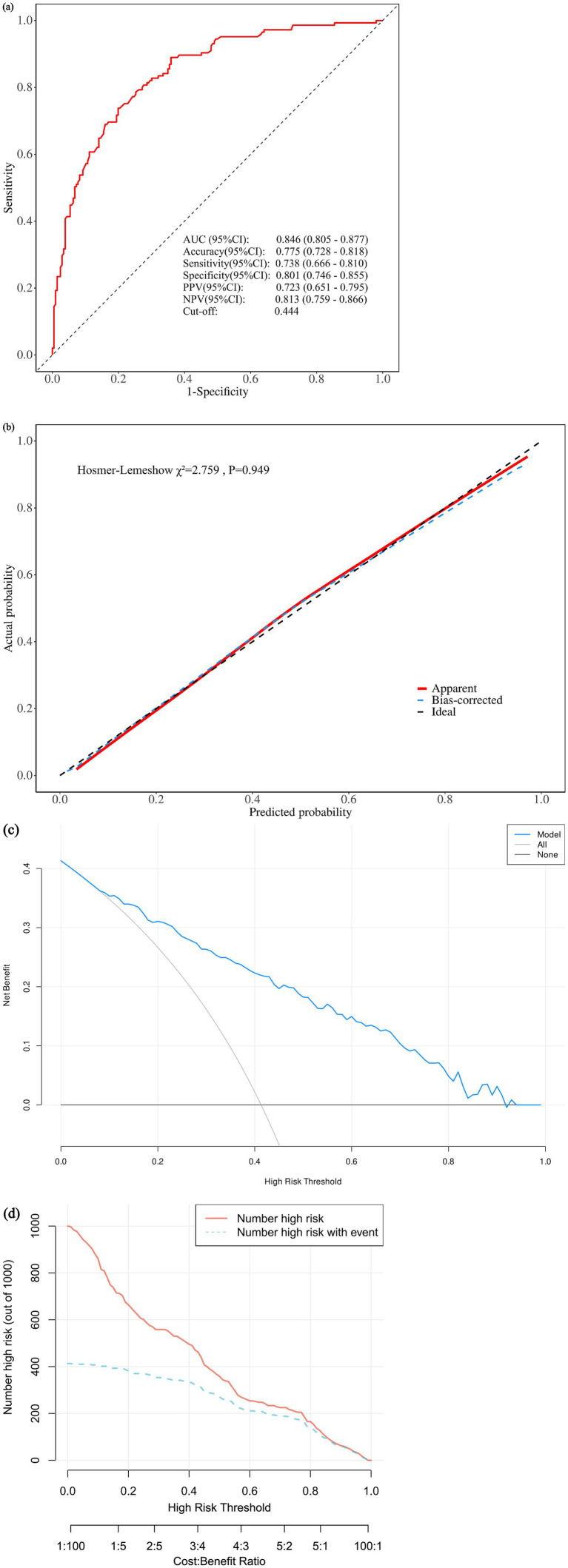
Validation of the nomogram prediction model. **(a)** Receiver operating characteristic (ROC) curve. The red solid line represents the model’s ROC curve; the black dashed diagonal line represents the reference line of no discrimination (AUC = 0.5). The area under the curve (AUC) is 0.846 (95% CI, 0.805–0.877), indicating good discriminatory ability. **(b)** Calibration plot. The *x*-axis shows the predicted probability of NTM-PD; the *y*-axis shows the actual probability. The black dashed line represents ideal calibration (perfect agreement). The red solid line represents apparent calibration, and the blue dashed line represents bias-corrected calibration by bootstrap resampling. The close alignment between the observed and ideal lines indicates excellent calibration (Hosmer–Lemeshow *χ*^2^ = 2.759, *p* = 0.949). **(c)** Decision curve analysis (DCA). The *y*-axis shows the net benefit; the *x*-axis shows the threshold probability for NTM-PD. The blue solid line represents the net benefit of using the model; the grey solid line represents the “treat-all” strategy; the black horizontal line represents the “treat-none” strategy (net benefit = 0). The model provides positive net benefit across a wide range of threshold probabilities (~5–85%), indicating clinical utility. **(d)** Clinical impact curve. The *y*-axis shows the number of patients classified as high risk per 1,000; the *x*-axis shows the threshold probability. The red solid line represents the number of patients classified as high risk by the model; the cyan dashed line represents the number of high-risk patients who actually experienced NTM-PD events. The close tracking between the two lines across most thresholds confirms the model’s practical value.

The discriminatory performance of the final model was assessed using the receiver operating characteristic curve. The area under the curve was 0.846 (95% confidence interval, 0.805 to 0.877) (Figure 5a). This indicates that the model has good discriminatory ability, with approximately 84.6% probability that a randomly selected NTM-PD patient will have a higher predicted risk score than a randomly selected PTB patient. At the optimal cut-off point determined by the Youden index (0.444), the model yielded a sensitivity of 73.8% and a specificity of 80.1%. This balance between sensitivity and specificity suggests that the model can correctly identify approximately three-quarters of NTM-PD cases while correctly ruling out four-fifths of PTB cases, making it a useful clinical screening tool before culture results become available. The Hosmer-Lemeshow goodness-of-fit test yielded a *χ*^2^ statistic of 2.759 (*p* = 0.949). The calibration plot visually presents the relationship between predicted probabilities and observed event frequencies across deciles of risk (Figure 5b). To internally validate the model and account for overfitting, the bootstrap resampling method was applied with 1,000 iterations. The optimism-corrected concordance index derived from this validation was 0.830.

The clinical utility of the model was analyzed using decision curve analysis (Figure 5c). Across a spectrum of threshold probabilities ranging from approximately 10 to 90%, the net benefit of the model-based decision strategy was plotted against the “treat-all” and “treat-none” strategies. A clinical impact curve was generated to compare the number of patients identified as high-risk by the model at various thresholds with the actual number of patients with NTM-PD (Figure 5d).

## 4 Discussion

The accurate and timely differentiation between NTM-PD and PTB remains a critical challenge in clinical practice, particularly in TB-endemic regions. In this study, we developed and internally validated a prediction model that effectively integrates key demographic, clinical, and radiographic characteristics to discriminate between these two diseases. The final model, comprising six readily accessible variables (Age, Gender, Diabetes mellitus, Bronchiectasis, COPD, and Lung cavitation), demonstrated robust discriminatory performance (AUC = 0.846) and good calibration. Notably, we employed LASSO regression for objective feature selection and rigorous bootstrap validation, enhancing the model’s reliability and potential for clinical translation.

Our findings align with and extend the existing literature on risk profiles for NTM-PD. The strong association between older age and NTM-PD corroborates numerous previous reports and likely reflects age-related decline in immune function and higher prevalence of underlying structural lung diseases (21, 22). Similarly, the identification of male gender as a protective factor (female gender as a risk factor for PTB) is consistent with epidemiological trends observed in several Asian cohorts, possibly linked to occupational exposures or immunological differences (23, 24). The absence of diabetes mellitus as a protective factor against NTM-PD, contrasting with its well-established role as a risk factor for PTB, represents a key differentiating point. This may be attributed to the distinct pathogenesis of the two infections; whereas hyperglycemia impairs cell-mediated immunity crucial for controlling *M. tuberculosis*, its impact on the host defense against typically less virulent NTM may be different or less pronounced (25).

Most strikingly, our model highlights the paramount importance of underlying structural lung disease and specific imaging phenotypes. The presence of bronchiectasis and COPD conferred high odds for NTM-PD (ORs of 5.44 and 4.31, respectively). These conditions disrupt mucociliary clearance and local immune defenses, creating a niche conducive to NTM colonization and infection (26). Furthermore, lung cavitation on HRCT emerged as the strongest independent predictor in our model (OR = 6.01). While cavitation is common in both diseases, our multivariate analysis suggests that its presence, particularly in the context of the other identified risk factors, significantly increases the probability of NTM-PD. This finding is biologically plausible given the NTM subspecies profile in our cohort, where *M. abscessus* (26.9%) and *M. kansasii* (19.3%)—both species frequently associated with cavitary disease—collectively accounted for nearly half of all cases (27, 28). The predominance of the *M. avium* complex (MAC, 46.2%), another common cause of cavitary NTM-PD, further supports the link between the imaging phenotype captured by our model and the prevalent pathogens in our setting (29).

Methodologically, our study addresses several limitations of prior prediction models. Unlike studies relying on univariate screening or conventional stepwise regression, we utilized LASSO regression to perform penalized feature selection from a broad set of candidate variables. This approach mitigates multicollinearity and overfitting, yielding a more parsimonious and stable model (30). The model’s excellent performance was further substantiated by bootstrap internal validation (optimism-corrected C-index = 0.830), providing a conservative estimate of its likely performance in similar populations. Finally, decision curve analysis confirmed the model’s clinical utility, demonstrating a positive net benefit across a wide range of threshold probabilities, thereby supporting its potential value in guiding diagnostic decisions when microbiological results are pending. The clinical relevance of our model is further reinforced by recent updates in diagnostic guidelines.

It is worth noting that after the completion of our study, the Chinese Society of Tuberculosis and the Chinese Thoracic Society jointly published an expert consensus on the diagnosis and treatment of NTM-PD complicated with bronchiectasis in 2025 (31). This consensus retains the core diagnostic principles from the 2020 guidelines (which align with ATS/IDSA criteria) but provides 14 evidence-based recommendations specifically for the management of NTM-PD when it coexists with bronchiectasis. Key recommendations relevant to our findings include: (1) bronchiectasis patients with new diagnoses, unexplained exacerbations, or those planning long-term macrolide therapy should be screened for NTM infection; and (2) the presence of cavitary lesions on imaging is an indication for initiating anti-NTM therapy. These recommendations are consistent with the predictors identified in our model—particularly bronchiectasis and lung cavitation—further supporting the clinical relevance of our findings. Future studies should consider integrating these updated consensus recommendations into the diagnostic workflow for suspected NTM-PD patients.

The clinical implications of our model are straightforward. For clinicians evaluating a patient with suspected mycobacterial infection, the presence of an older female patient without diabetes but with a history of bronchiectasis or COPD, whose HRCT reveals cavitary lesions, should strongly raise the suspicion for NTM-PD over PTB. This recommendation is supported by our model’s findings: each of these factors was independently associated with NTM-PD in multivariable analysis (Table 4), with odds ratios ranging from 4.31 (COPD) to 6.01 (lung cavitation) (all *p* < 0.001). The combination of these features yields a high predicted probability of NTM-PD, as visualized in the nomogram (Figure 4). Furthermore, the predominance of cavitation-associated NTM subspecies in our cohort—*M. abscessus* (26.9%) and *M. kansasii* (19.3%), both known to cause cavitary disease—provides microbiological support for this imaging-based predictor (Table 2). This pre-culture risk assessment can prompt more targeted diagnostic testing (e.g., requesting NTM-specific cultures or molecular tests) and prevent the initiation of inappropriate, potentially toxic anti-tuberculosis therapy.

Our study has several limitations that must be acknowledged. First, its single-center, retrospective design may introduce selection bias. All patients were hospitalized, likely representing a more severe spectrum of disease, which may limit the generalizability of our model to outpatient or milder cases. The model’s performance in primary care or outpatient settings, where disease prevalence and severity differ, requires further evaluation. Second, while internal validation via bootstrap is a strength, external validation in independent, multicenter cohorts—ideally from different geographic regions—is essential to confirm the model’s transportability and performance across diverse healthcare settings. Third, the diagnosis of comorbidities like bronchiectasis and COPD relied on clinical documentation and available imaging, which may lack the granularity of prospective, protocol-defined assessments. Fourth, the model was developed to distinguish between two specific diseases (NTM-PD vs. PTB); its performance in a broader differential diagnosis including other cavitary lung diseases (e.g., fungal infections, lung cancer) requires further evaluation. Finally, while we reported the distribution of major NTM species (predominantly MAC, *M. abscessus*, and *M. kansasii*), our sample size was insufficient to develop subtype-specific models, which may have distinct clinical-radiographic signatures.

In conclusion, we developed and internally validated a parsimonious clinical prediction model that effectively differentiates NTM-PD from PTB using six readily available variables objectively selected by LASSO regression: age, gender, diabetes mellitus, bronchiectasis, COPD, and lung cavitation. The model demonstrated good discriminatory ability (AUC = 0.846, 95% CI: 0.805–0.877), excellent calibration (Hosmer-Lemeshow test, *p* = 0.949), and clinical utility across a wide range of threshold probabilities in decision curve analysis, with internal validation confirming its stability (optimism-corrected C-index = 0.830). This model can serve as a practical pre-culture screening tool to help clinicians identify patients with a high probability of NTM-PD and prompt targeted diagnostic testing, thereby reducing misdiagnosis and inappropriate anti-tuberculosis therapy. However, before widespread clinical implementation, external validation in independent, multicenter cohorts is essential. Future studies should also explore subspecies-specific models and integrate emerging biomarkers to further refine predictive accuracy.

## Data Availability

The original contributions presented in the study are included in the article/supplementary material, further inquiries can be directed to the corresponding author.
